# Predicting Progression of IgA Nephropathy: New Clinical Progression Risk Score

**DOI:** 10.1371/journal.pone.0038904

**Published:** 2012-06-14

**Authors:** Jingyuan Xie, Krzysztof Kiryluk, Weiming Wang, Zhaohui Wang, Shanmai Guo, Pingyan Shen, Hong Ren, Xiaoxia Pan, Xiaonong Chen, Wen Zhang, Xiao Li, Hao Shi, Yifu Li, Ali G. Gharavi, Nan Chen

**Affiliations:** 1 Nephrology Department, Ruijin Hospital, Shanghai Jiao Tong University School of Medicine, Shanghai, China; 2 Division of Nephrology, Department of Medicine, College of Physicians and Surgeons, Columbia University, New York, New York, United States of America; Institut national de la santé et de la recherche médicale (INSERM), France

## Abstract

IgA nephropathy (IgAN) is a common cause of end-stage renal disease (ESRD) in Asia. In this study, based on a large cohort of Chinese patients with IgAN, we aim to identify independent predictive factors associated with disease progression to ESRD. We collected retrospective clinical data and renal outcomes on 619 biopsy-diagnosed IgAN patients with a mean follow-up time of 41.3 months. In total, 67 individuals reached the study endpoint defined by occurrence of ESRD necessitating renal replacement therapy. In the fully adjusted Cox proportional hazards model, there were four baseline variables with a significant independent effect on the risk of ESRD. These included: eGFR [HR = 0.96(0.95–0.97)], serum albumin [HR = 0.47(0.32–0.68)], hemoglobin [HR = 0.79(0.72–0.88)], and SBP [HR = 1.02(1.00–1.03)]. Based on these observations, we developed a 4-variable equation of a clinical risk score for disease progression. Our risk score explained nearly 22% of the total variance in the primary outcome. Survival ROC curves revealed that the risk score provided improved prediction of ESRD at 24th, 60th and 120th month of follow-up compared to the three previously proposed risk scores. In summary, our data indicate that IgAN patients with higher systolic blood pressure, lower eGFR, hemoglobin, and albumin levels at baseline are at a greatest risk of progression to ESRD. The new progression risk score calculated based on these four baseline variables offers a simple clinical tool for risk stratification.

## Introduction

IgA nephropathy (IgAN) is the most common form of primary glomerulonephritis (GN) worldwide [1]. The disease is characterized by a highly variable clinical course ranging from a benign condition to a rapidly progressive irreversible kidney failure. About 15 to 40 percent of IgAN patients will develop worsening renal dysfunction and eventually end stage renal disease (ESRD) within 10–20 years of diagnosis [2], [3], [4]. A major challenge in the field is the identification of individuals at highest risk of progression to ESRD. Notably, IgAN is most prevalent in Asia, and studies suggest that the disease may have a more severe course in individuals of Asian ancestry [5], [6]. Thus, studies based on Asian populations may be more effective in identifying risk factors for progression.

Numerous prior studies identified several potential clinical predictors of progression, including degree of renal impairment at diagnosis [2], [7], [8], [9], histologic grading [2], [7], [8], [9], [10] and proteinuria [10], [11], [12]_ENREF_8. These factors appear to contribute independently to the risk of progression in multivariate models. Moreover, some studies suggest an independent prognostic value of high blood pressure at presentation [6], [7], [10] ?r during follow-up [12], hematuria [9], family history of hypertension [7] or chronic renal failure [9], serum albumin level [9], [13], age [9], and male gender [9].

One of the problems in the field is that several of the above predictors reflect the degree of disease severity on presentation and are thus strongly inter-correlated. Their individual contribution to the overall risk of progression is difficult to assess without powerful and well-characterized patient cohorts. In addition, the overall predictive value of these variables is relatively low. The development of a risk score that reflects cumulative effects of individual predictors may be helpful to identify individuals that are most likely to progress to ESRD. This approach has been successfully utilized in the RENAAL study of 1,513 type 2 diabetics with nephropathy [14]. Based on the longitudinal data from this study, a relatively simple risk score was proposed that incorporates serum creatinine, albumin, hemoglobin, and urine albumin-to-creatinine ratio into an equation that accurately determines the risk of progression to ESRD. Another powerful example of this approach is provided by a large-scale progression study of all-cause chronic kidney disease (CKD) [15]. Here, the most accurate model that predicted ESRD included age, sex, eGFR, albuminuria, as well as basic serum measurements of calcium, phosphate, bicarbonate, and albumin. This model was further validated in an independent cohort of 4,942 patients with an estimated C-statistic of 0.84 (95%CI 0.83–0.86).

To date, there have been two studies that applied a similar approach to the prediction of ESRD in patients with newly diagnosed IgAN: the study by Berthoux et al. of 332 French patients followed for a median of 136 months [10] and the study by Goto et al. of 2,283 Japanese patients followed for a median of 87 months [9]. The French study derived a 3-variable risk score (based on hypertension, proteinuria, and a histology score), while the Japanese study derived an 8-variable score (based on age, gender, hypertension, proteinuria, hematuria, hypoalbuminaemia, eGFR, and histological grade). The performance of these risk scores, however, has not yet been validated in independent cohorts.

In this study, we systematically evaluate the predictive value of a complete set of baseline clinical and laboratory factors in the progression of renal disease in a large cohort of IgAN patients from Shanghai, China. We formulate a new 4-variable risk score equation that best predicts renal disease progression in our cohort. We also compare the performance of our Risk Score to the French and the Japanese progression scores, as well as to the risk score based on the RENAAL study.

## Methods

### Ethics Statement

This study was approved by the Institutional Review Board of the Ruijin Hospital, Shanghai Jiao Tong University School of Medicine and was in accordance with the principle of the Helsinki Declaration II. The written informed consent was obtained from each participant.

### Study Population and Clinical Data

To identify cases eligible to participate in the study, we reviewed all kidney biopsy reports of Shanghai Jiao Tong University Ruijin Hospital, a major referral center for Southern China, between years 1989 and 2010. All cases included in the study were defined by dominant and at least 2+ (on a scale from 0 to 3+) mesangial staining for IgA by immunoflorescence, in addition to compatible findings of mesangial expansion or proliferation on light microscopy. The patients with systemic diseases, such as systemic lupus erythematosus, Henoch-Schonlein purpura, and chronic liver disease were excluded from this analysis. We also recruited a large group of age and gender matched healthy controls from the Ruijin Hospital Health Care Center defined by the absence of abnormalities on a routine urinalysis and normal renal function (serum creatinine <1.1 mg/dL).

In total, 619 individuals fulfilled our diagnostic criteria for IgAN and had a minimum of 3 months of follow-up data available for analysis. Baseline demographic and clinical data were collected from all patients at the time of renal biopsy. These included: age, gender, body mass index (BMI), serum creatinine, serum uric acid (UA), triglyceride levels, cholesterol levels, hemoglobin (Hb), systolic blood pressure (SBP), diastolic blood pressure (DBP), family history of kidney disease, history of gross hematuria, serum immunoglobulin A (IgA) levels, and 24-hour protein excretion. The diagnosis of hypertension was based on SBP≥140 mmHg, or DBP≥90 mmHg, or history of antihypertensive medication use. Hyperuricemia was defined by gender-specific criteria of serum UA >450 umol/L in males and >340 umol/L in females. Anemia was defined by gender-specific criteria of hemoglobin concentrations <13.5 g/dL in males and <12 g/dL in females. Hypoalbuminemia was defined by serum albumin <3 g/dL. Estimated glomerular filtration rate (eGFR) was evaluated by an abbreviated Modification of Diet in Renal Disease (MDRD) equation modified for Chinese: eGFR (ml/min/1.73 m^2^)  = 186*Pcr ^−1^.^154^*age ^−0^.^203^*0.742(if female)*1.233 [16]. Chronic kidney disease (CKD) was classified based to the Kidney Disease Outcomes Quality Initiative (K/DOQI) practice guidelines [17]. Most patients were treated according to the accepted standards at our center: IgAN patients with hypertension and/or proteinuria were treated with ACE inhibitors (ACEI) and/or angiotensin receptor blockers (ARB). Glucocortoids were added in individuals with a new onset of massive proteinuria, and proteinuric patients who did not respond to an ACEI or ARB therapy. Patients with crescentic disease and rapidly progressive glomerulonephritis were treated with combined immunosuppressive agents and glucocorticoids. The mean follow-up time after renal biopsy was 41.3 months (range 3.03–248.1 months).

### Statistical Methods

The distributions of quantitative variables were assessed for normality and summarized as means and standard deviations (or medians and ranges for non-normally distributed variables). Statistical testing of continuous variables was performed using Student’s t-test (or Mann-Whitney U-test if appropriate). All categorical variables were expressed as frequencies or percentages (%) and comparison of proportions was performed using a standard X^2^ test. Baseline clinical variables included sex, age, family history, BMI, baseline serum creatinine, eGFR, SBP, DBP, mean arterial pressure, pulse pressure, urine protein, gross hematuria, serum UA, serum albumin, serum triglycerides, serum cholesterol, hemoglobin, platelets, WBC, serum IgA, Haas classification, and treatment type. All slides of kidney biopsies were reviewed by a single experienced renal pathologist. The primary outcome was defined as occurrence of ESRD defined by a need for renal replacement therapy (dialysis or renal transplantation). The association of baseline variables with the primary outcome was tested using Cox regression proportional hazards models. A two sided P<0.05 was considered statistically significant. To identify independent predictors of progression, we performed a multivariate Cox regression analysis with a stepwise selection of variables (entry and elimination P<0.05). Patients were censored at the time of death or loss to follow-up. The proportional hazards assumption was formally tested for each of the outcomes using the method proposed by Grambsch and Therneau [18] and implemented in the R survival package version 2.36 (R v.2.9). The independent predictors retained in the final model were used to derive the Risk Score. The effects of each independent predictor, as well as their cumulative effect in the form of the Risk Score were next tested using the Kaplan-Meier approach. We also scored our patients using the Japanese [9], the French [10] and the RENAAL [14] risk scores. The R^2^ (reflecting the fraction of variance in the primary outcome explained) was determined for each of the models [19]. In addition, survival areas under receiver operating characteristic (ROC) curves were also assessed for the 24^th^, 60^th^ and 120^th^ month time points. These analyses were performed using Survcomp [20] package version 1.1.6 (R v.2.9) and ROCR package version 1.0–2 (R v.2.9) [21]. Based on the size and median follow-up of our cohort, we estimate 80% power to detect hazard ratios greater than 1.4 in this study. Our power calculations were performed with the PS software version 3.0 [22].

## Results

### Baseline Demographic and Clinical Data

We analyzed clinical data from a total of 619 IgAN patients (Table 1). There were 314 males and 305 females in the study; the average age was 36±12 years. Of all the IgAN patients, 78 (12.6%) had positive family history of chronic kidney disease. Most IgAN patients had moderate to severe pathology grade at diagnosis (75.4% with Haas III-V). Moreover, 46.9% were hypertensive, 61.7% had urine protein higher than 1 g/24 h, and 20.2% reported history of gross hematuria (Table 1). We first explored the associations of clinical variables with baseline renal function (eGFR). In univariate analyses, 17 of 25 baseline clinical variables correlated with the degree of renal impairment at the time of biopsy (Table S1). In multivariate analysis, older age [beta = −1.06, p<2.0*10^−16^], higher degree of proteinuria [beta = −5.18, p = 2*10^−3^], elevated UA [beta = −8.23, p<2.0*10^−16^], higher Haas grade [beta = −11.3, p = <2.0*10^−16^), lower hemoglobin, [beta = 2.90, p = 9*10^−7^] and increased SBP [beta = −0.37, p = 2*10^−7^] were independently associated with lower eGFR at the time of biopsy (Table S2).

**Table 1:** Baseline characteristics of IgAN patients.

Variable	IgAN Patients, N = 619
Follow-up mean (scope), [month]	41.3 (3.03–248.1)
Age at biopsy (±s.d.), [years]	36.0±12.3
Gender (Male: Female)	1.03∶1.00
Family history of chronic kidney disease (%)	78 (12.6)
Body mass index mean (±s.d.), [kg/m^2^]	23.0±3.5
Serum creatinine mean (±s.d.), [mg/dL]	1.5±1.1
GFR mean (±s.d.), [mL/min/1.73 m^2^]	87.9±44.4
CKD stage 1 (%)	289 (46.7)
CKD stage 2 (%)	135 (21.8)
CKD stage 3 (%)	145 (23.4)
CKD stage 4 (%)	38 (6.1)
CKD stage 5 (%)	12 (1.9)
SBP mean (±s.d.), [mm Hg]	128.2±18.9
DBP mean (±s.d.), [mm Hg]	82.5±13.0
MAP mean (±s.d.), [mm Hg]	97.7±14.1
Pulse pressure mean (±s.d.), [mm Hg]	45.7±12.1
Hypertension (%)	290 (46.9)
Urine protein median (scope), [g/24 h]	1.42 (0–13.9)
Urine protein groups	
Mild (<1 g/24 h) (%)	237 (38.3)
Moderate (1∼3 g/24 h) (%)	254 (41)
Severe (> = 3 g/24 h) (%)	128 (20.7)
Gross hematuria (%)	125 (20.2)
Serum UA mean (±s.d.), [mg/dl]	6.5±1.7
Hyperuricemia (%)	256 (41.4)
Serum albumin mean (±s.d.), [g/dL]	3.4±0.8
Hypoalbuminemia (%)	128 (20.7)
Serum triglycerides median (scope), [mg/dL]	175.7(42.5–1033.6)
Serum cholesterol median (scope), [mg/dL]	198.1(32.1–469.1)
Hemoglobin mean (±s.d.), [g/dl]	13.0±2.2
Anemia (%)	261 (42.2)
WBC mean (±s.d.), [10^3^/mm^3^]	7.7±2.7
Serum IgA mean (±s.d.), [mg/L]	3238.5±1422.9
Haas classification	
Grade I (%)	16(2.6)
Grade II (%)	136(22)
Grade III (%)	251(40.5)
Grade IV (%)	130(21)
Grade V (%)	86(13.9)
ACEI or ARB treatment (%)	368(67.8%)
Glucocorticoid treatment (%)	293(54.7)

SBP: systolic blood pressure;

DBP: diastolic blood pressure;

MAP: mean arterial pressure;

UA: uric acid;

WBC: white blood cell count.

### Predictors of Progression in IgAN

In total, 67 individuals reached the study endpoint defined as occurrence of ESRD. In univariate analyses, 21 of 30 baseline variables were significantly associated with this outcome (Table 2). In the multivariate stepwise Cox proportional hazards models, only four baseline variables had a significant, independent effect on the risk of ESRD (Model 1): baseline eGFR [HR = 0.96, 95% CI 0.95–0.97, p = 1.3*10^−14^], serum albumin [HR = 0.47, 95% CI 0.32–0.68, p = 7.4*10^−5^], hemoglobin [HR = 0.79, 95% CI 0.72–0.88, p = 1.2*10^−5^], and SBP [HR = 1.02, 95% CI 1.00–1.03, p = 5.4*10^−3^] (Table 3). Similarly, the same four variables were also highly significant independent predictors of eGFR decline (defined as 50% reduction from the baseline eGFR) in our cohort (Model 2): baseline eGFR [HR = 0.98, 95% CI 0.98–0.99, p = 1.6*10^−8^], serum albumin [HR = 0.46, 95% CI 0.35–0.62, p = 2.7*10^−7^], hemoglobin [HR = 0.81, 95% CI 0.75–0.89, p = 1.5*10^−6^], and SBP [HR = 1.02, 95% CI 1.00–1.03, p = 6.4*10^−3^]. We also explored all pairwise interactions and considered quadratic terms in these models, but none these alternative analyses provided a better fit to the data.

**Table 2:** Univariate analysis of baseline variables with renal end points for ESRD.

	IgAN Patients, N = 619		
Predictor	HR	95% CI	P value
Age at biopsy [year]	1.02^#^	1.01–1.04	4*10^−2^
Female Gender	0.69	0.42–1.13	0.14
Family history	0.75	0.37–1.52	0.42
Body mass index [kg/m^2^]	0.94	0.82–1.09	0.41
Serum creatinine [mg/dL]	2.30^#^	2.03–2.61	<2.0*10^−16^
eGFR [mL/min/1.73 m^2^]	0.95^#^	0.94–0.96	<2.0*10^−16^
eGFR [<60 to > = 60 mL/min/1.73 m^2^]	7.91^#^	4.60–13.6	7.6*10^−14^
SBP [mm Hg]	1.03^#^	1.02–1.04	1.2*10^−7^
SBP [> = 140 to <140 mmHg]	2.85^#^	1.76–4.63	2.3*10^−5^
DBP [mm Hg]	1.04^#^	1.02–1.06	7.5*10^−6^
DBP [> = 90 to <90 mmHg]	2.90^#^	1.76–4.77	2.9*10^−5^
MAP [mm Hg]	1.04^#^	1.03–1.06	3.6*10^−7^
Pulse pressure [mm Hg]	1.03^#^	1.01–1.05	5.5*10^−4^
Hypertension	3.59^#^	2.07–6.23	5.7*10^−6^
Urine protein [g/24 h]	1.12^#^	1.03–1.21	6.9*10^−3^
Degree of proteinuria [per group]	2.28^#^	1.64–3.18	1.0*10^−6^
Gross hematuria	0.57	0.31–1.06	0.07
Serum UA [mg/dl]	1.40^#^	1.23–1.60	6.1*10^−7^
Hyperuricemia	2.11^#^	1.29–3.45	2.9*10^−3^
Serum albumin [g/dL]	0.60^#^	0.45–0.79	2.3*10^−4^
Hypoalbuminemia	2.45^#^	1.48–4.07	5.0*10^−4^
Serum triglycerides [mg/dL]	1.00	0.99–1.00	0.64
Serum cholesterol [mg/dL]	1.00	0.99–1.01	0.15
Hemoglobin [g/dl]	0.75^#^	0.69–0.82	2.0*10^−11^
Anemia	4.98^#^	2.80–8.85	4.8*10^−8^
WBC [10^3^/mm^3^]	0.90	0.72–1.13	0.38
Serum IgA [mg/L]	1.00	0.99–1.00	0.99
Haas classification	2.71^#^	2.05–3.57	2.1*10^−12^
ACEI or ARB treatment (%)	0.71	0.42–1.20	0.21
Glucocorticoid treatment	1.52	0.95–2.40	0.10

SBP: systolic blood pressure;

DBP: diastolic blood pressure;

MAP: mean arterial pressure;

UA: uric acid;

WBC: white blood cell count.

# p<0.05.

**Table 3:** Multivariate Cox Regression with Stepwise Selection (n = 619).

Variable	Coefficient	HR (95%CI)	R^2^ (%)	P-value
Model 1* (events = 67)				
eGFR [ml/min/1.73 m^2^]	−0.039	0.96 (0.95–0.97)	16.3	1.3*10^−14^
Hemoglobin [g/dL]	−0.230	0.79 (0.72–0.88)	6.4	1.2*10^−5^
Serum albumin [g/dL]	−0.762	0.47 (0.32–0.68)	1.9	7.4*10^−5^
SBP [mmHg]	0.016	1.02 (1.00–1.03)	3.7	5.4*10^−3^
Risk Score #		2.73 (2.27–3.28)	21.9	<2*10^−16^
**Model 2** ** **(events = 85)**				
eGFR [ml/min/1.73 m^2^]	−0.018	0.98 (0.98–0.99)	7.5	1.6*10^−8^
Hemoglobin [g/dL]	−0.206	0.81 (0.75–0.89)	6.5	1.5*10^−6^
Serum albumin [g/dL]	−0.769	0.46 (0.35–0.62)	3.2	2.7*10^−7^
SBP [mmHg]	0.015	1.02 (1.00–1.03)	3.0	6.4*10^−3^
Risk Score #		1.78 (1.56–2.02)	14.0	<2*10^−16^

SBP: systolic blood pressure.

* Renal outcome defined as end-stage renal disease (ESRD).

** Renal outcome defined as 50% decline from baseline eGFR.

# The risk score was calculated from the coefficients of independent risk factors in model 1.

**Figure 1:**
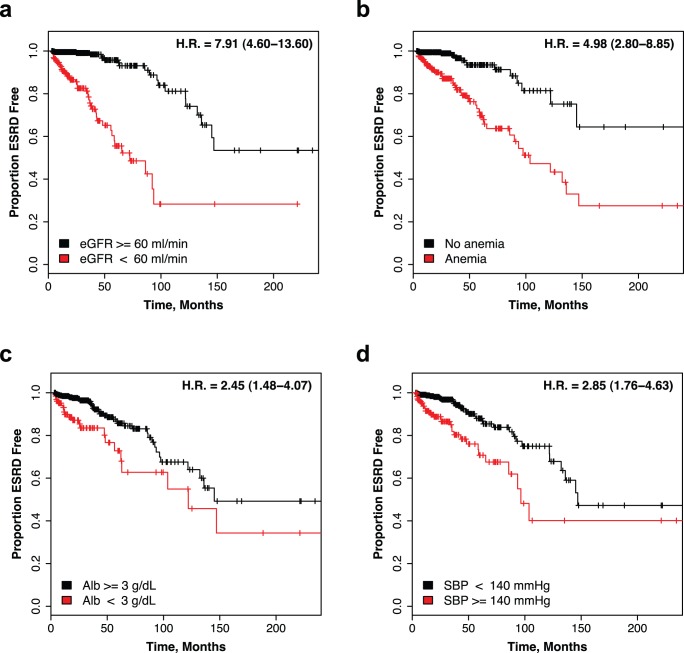
Kaplan-Meier Outcome-free Survival Curves. **(a)** low (red) versus high (black) baseline eGFR group; **(b)** patients with a baseline diagnosis of anemia (red) versus no anemia (black); **(c)** patients with hypoalbuminemia (red) versus normoalbuminemia (black); **(d)** patients with systolic hypertension (red) versus normotensives (black). Censor points are denoted by vertical tick lines.

As expected, eGFR at presentation was the strongest predictor of ESRD: each unit decrease in baseline eGFR was associated with 4% increase in the risk of ESRD during the follow-up period. Accordingly, individuals with baseline eGFR >60 ml/min/1.73 m^2^ had considerably longer median outcome-free survival time when compared to those with eGFR <60 ml/min/1.73 m^2^ (242 months versus 72 months) [HR = 7.91, 95%CI: 4.60–13.60, Figure 1A].

On average, the cases were three times more likely to fulfill our diagnostic criteria for anemia compared to the healthy population controls (42.2% vs. 14.5%) (Table S3). Each unit drop in hemoglobin was associated with 20% increase in the risk of ESRD. Median outcome-free survival times were 104 and 247 months in individuals with and without the diagnosis of anemia, respectively [HR = 4.98, 95% CI 2.80–8.85, Figure 1B]. In addition to the risk of ESRD, anemia was associated with male sex, older age, lower eGFR, higher SBP, more severe proteinuria, higher uric acid level, lower albumin level, and more severe Haas class (Table S4). Individuals with low hemoglobin levels were also more frequently treated with glucocorticoids.

**Figure 2:**
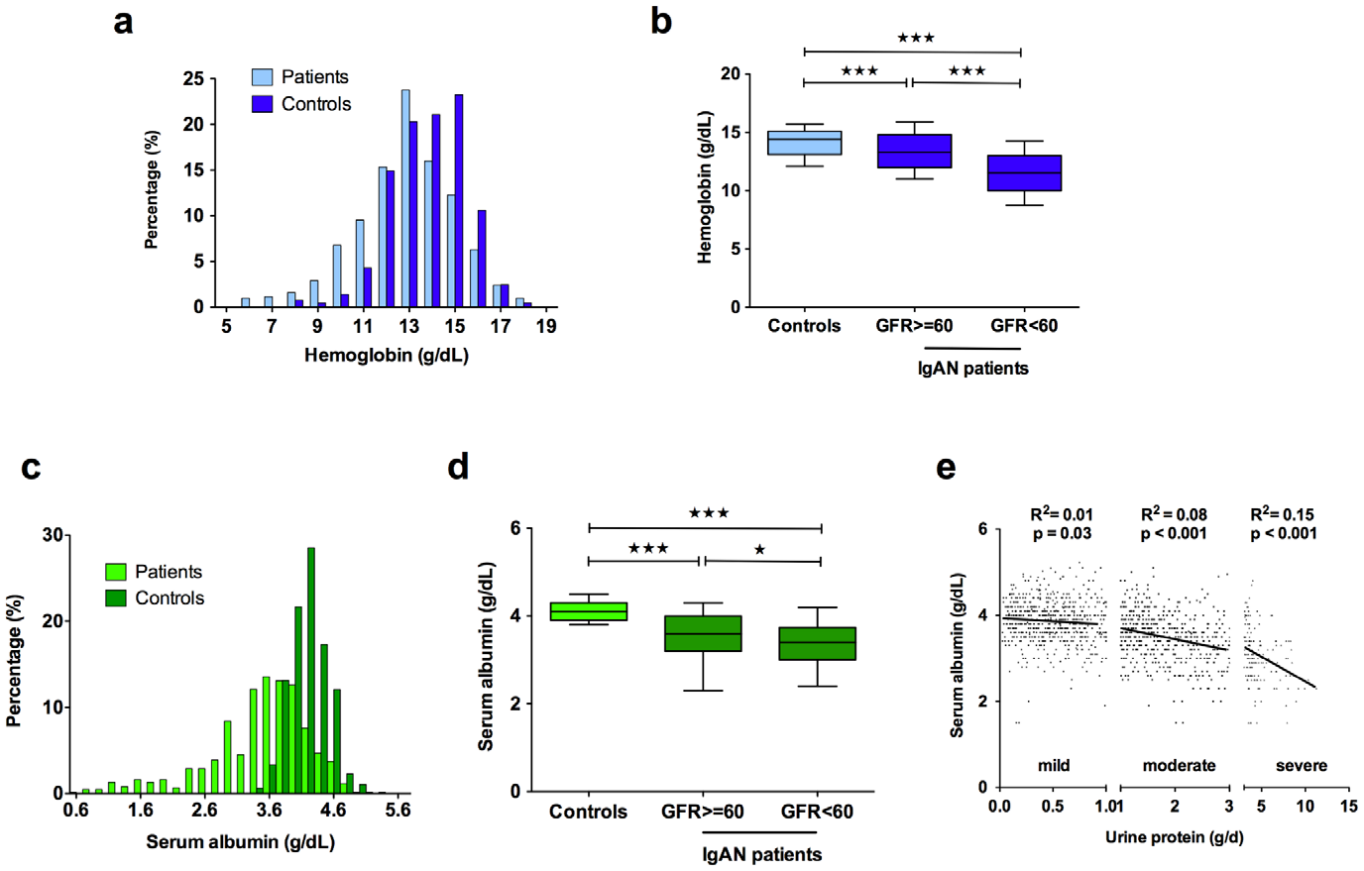
Detailed Analysis of Hemoglobin and Serum Albumin Levels. (a) the distributions of hemoglobin levels for IgAN patients and healthy controls; **(b)** hemoglobin levels by the degree of renal impairment; **(c)** serum albumin distributions in IgAN patients and healthy controls; **(d)** serum albumin levels by the degree of renal impairment; **(e)** correlation between serum albumin and urine protein excretion by three different groups of proteinuria. Significance code: * p<0.05, ** p<0.01, *** p<0.001.

The patients with hypoalbuminemia had a shorter median ESRD-free survival of 122 months, compared to 145 months for those with normal albumin levels [HR = 2.45, 95% CI 1.48–4.07, Figure 1C]. Serum albumin was strongly correlated with daily protein excretion (Figure 2C-E, Table S4). Surprisingly, proteinuria did not independently contribute to the risk of ESRD in multivariate analysis. We formally explored if albumin and/or hemoglobin account for the effect of proteinuria in the final risk model (Table S5). The exclusion of albumin from the full model unmasked highly significant association of proteinuria with the risk of progression (HR 1.56, p = 9.7×10^−3^), but at the cost of overall reduction in the model’s goodness of fit. This suggests that albumin is a superior predictor of outcome and captures most of the variance contributed by proteinuria.

### Progression Risk Score

**Figure 3:**
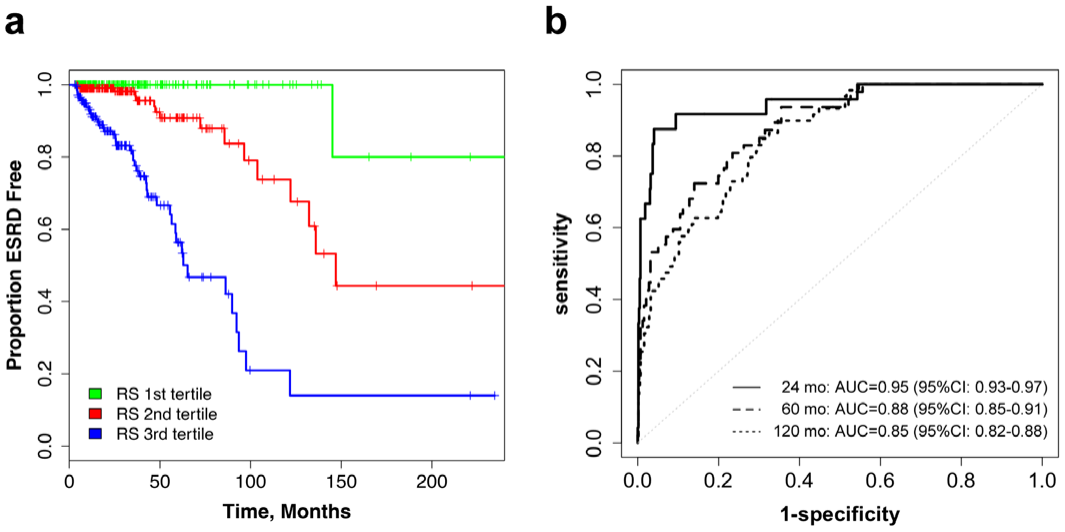
Survival and Survival ROC curves for the Risk Score. (a) Kaplan-Meier outcome-free survival curves by risk score tertiles; **(b)** the Risk Score’s ROC curves for predicting ESRD at 24 months, 60 months and 120 months.

Next we developed a risk score for disease progression based on the regression coefficients for the four independent predictors retained in the best model (Table 3). The risk score equation is provided by the following formula:


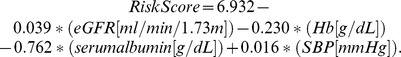
When considered in a stepwise multivariate analysis with all 21 other baseline variables at entry, the risk score was the only independent predictor of adverse renal outcome. It conveyed 2.7-fold increase in the risk of ESRD per one score unit [HR = 2.73, 95%CI: 2.27–3.28] and explained 21.9% of the total variance in the primary outcome. The median ESRD-free survival times for the lowest, middle, and highest tertiles of the Risk Score were 247, 147, and 65 months, respectively. Accordingly, when compared to the first tertile, individuals in the second Risk Score tertile had a 15-fold increase in the risk of ESRD [HR = 15.3, 95%CI: 2.0–115.0)], while individuals in the highest tertile had over 79-fold risk increase [HR = 79.8, 95%CI 11.0–580.3] (Figure 3A and Table S6).

Survival ROC analysis revealed that the risk score provided considerably improved discriminative power at 24, 60 and 120 months of follow-up compared to individual predictors. The area under the survival ROC curves was estimated at 0.95 (95%CI: 0.93–0.97) at 24 months, 0.88 (95% CI: 0.85–0.91) at 60 months, and 0.85 (95%CI: 0.82–0.88) at 120 months of follow-up. Impressively, at the cutoff point of 3.27, the Risk Score’s sensitivity and specificity of predicting ESRD within 2 years of diagnosis were 87.5% and 96.0%, respectively (Figure 3B).

**Figure 4:**
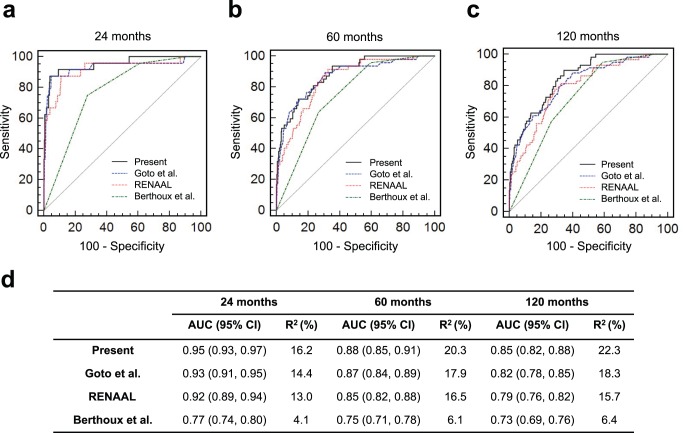
Performance of the Published ESRD Prediction Scores. The ROC curves for predicting renal outcomes within (a) 24 months, (b) 60 months, and (c) 120 months of follow-up. The Risk Score from this study (black) is contrasted against the Goto et al. score (blue), RENAAL score (red) and the Berthoux et al. score (green); (d) comparisons of AUCs (and their 95% CIs) and R^2^ for the four risk score prediction models.

Next, we compared the performance of the three other published risk scores in predicting ESRD in our dataset (Figure 4). The Goto et al. Japanese progression score performed better compared to the Berthoux et al. and the RENAAL scores, with the AUC of 0.93, 0.87 and 0.82 at 24, 60, and 120 months of follow-up, respectively. This score explained 14.4%, 17.9% and 18.3% of variance in the primary outcome for each respective follow-up period. The performance of the Goto et al. score was only slightly worse compared to the Risk Score derived in our study (AUC of 0.95, 0.88, and 0.85; variance explained: 16.2%, 20.3% and 22.3%). The RENAAL risk score provided slightly less accurate prediction compared to the Goto et al. score, with respective AUCs of 0.92, 0.85, and 0.79. These differences in performance are likely due to the fact that this score was originally derived for patients with diabetic nephropathy. Finally, the risk score proposed by Berthoux et al. was considerably less accurate, with respective AUCs of 0.77, 0.75 and 0.73.

## Discussion

IgAN is a progressive disease with high variability of clinical presentation and outcomes [23]. Presently, clinician’s ability to identify patients at a highest risk of progression is limited. Such patients, however, are more likely to benefit from early or more aggressive therapy. In this study, we systematically test a complete set of over 28 baseline clinical parameters in multivariate models to detect independent predictors of renal disease progression. This is one of the largest observational studies of IgAN, involving over 600 patients. Accordingly, we are well powered to detect relatively small effect sizes. Other strengths of our study include: homogenous patient cohort, uniform histology scoring of renal biopsies, and application of a robust definition of progression (ESRD requiring renal replacement therapy).

To our knowledge, our study is the first to identify hemoglobin level as an independent risk factor for progression of IgAN. In the adjusted models, each 1 g/dL drop in hemoglobin was associated with 20% increase in the risk of renal progression. Additionally, hemoglobin levels explained nearly 6.4% of variance in renal outcome. Anemia is a common complication of CKD that has recently emerged as an important independent risk factor for kidney disease progression [24], [25]. For example, in the RENNAL cohort, baseline hemoglobin concentration was inversely correlated with the risk of ESRD, with the average increase in the risk of 11% per each 1 g/dL decline in hemoglobin levels after adjustment for baseline renal function and other covariates. The exact mechanism that underlies these observations is not clear. It is possible that anemia has a direct causal effect on the deterioration of renal function. Alternatively, this association may reflect the severity of underlying systemic inflammation, or may mark additional kidney damage that is not yet reflected by a decline in eGFR.

In addition to hemoglobin levels, our study provides strong support for predictive value of serum albumin in the assessment of ESRD risk. Serum albumin is widely recognized as a biomarker of nutritional status and inflammation, but it is also closely correlated with age, proteinuria, and hemoglobin levels (Figure 3C-D, Table S4). Multiple prior studies have found independent associations of low serum albumin with disease progression outcomes among patients with diabetic nephropathy and CKD [14], [15], [25], [26], [27], [28]. Thus, similar to hemoglobin levels, our study contributes to the growing evidence for hypoalbuminemia as a major risk factor for ESRD and validates its utility in patients with IgA nephropathy.

Our findings also confirm strong independent associations of decreased eGFR, and elevated SBP with accelerated renal disease progression. These clinical parameters are among the most consistently reported predictors of progression, with similar findings observed across multiple cohorts [29], [30], [31], [32].

Interestingly, proteinuria was strongly associated with the risk of ESRD in univariate analysis, however, it did not independently contribute to the risk in multivariate models. Notably, urinary protein had strong inverse correlation with serum albumin. Accordingly, inclusion of albumin in the prediction model captured most of the variance in outcome contributed by proteinuria. Although albumin appears to be a superior predictor of progression in our cohort, it is also possible that additional predictive value of proteinuria would become more evident with larger cohort size or longer follow-up.

Based on our results, we formulated a new four-variable risk score model for predicting ESRD. Our Risk Score explained nearly 22% of the total variance in the outcome. In addition, when tested against the three previously proposed scores, our Risk Score provided improved prediction of ESRD at 24^th^, 60^th^ and 120^th^ month of follow-up.

Previously, the largest and most comprehensive IgAN progression study with a similar endpoint of ESRD was performed in Japanese individuals by Goto et al. [9] This nation-wide study followed 2,283 IgAN patients from 97 clinical units in Japan for a median of 87 months with the primary outcome of ESRD. The study formulated an 8-variable progression score that included age, gender, hypertension, proteinuria, hematuria, hypoalbuminaemia, eGFR, and histological grade. In our study, we provide the first independent validation of this risk score. However, our Risk Score had better discrimination power despite comprising of a smaller number of variables. It is noteworthy that the Japanese study did not consider hemoglobin levels and/or anemia diagnosis as potential predictors of ESRD. Based on our findings, the addition of anemia would significantly strengthen their model.

Other risk scoring systems, such as the Bethoux et al. [10] or the Bartosik et al. [33] are not directly comparable to our risk score because they did not examine hemoglobin, serum albumin, or other baseline laboratory measurements. The Bethoux’s formula incorporates proteinuria, hypertension, and histology score, while the Bartosik’s formula includes mean arterial pressure and proteinuria, but requires follow-up data of at least 2 years. Neither of these risk scores uses the generally accepted Haas or Oxford classification systems. Not surprisingly, the Berthoux risk score did not perform well in predicting ESRD in our dataset. Although the Bartosik formula was validated by another small cohort [34], it uses a less definitive clinical outcome (slope of eGFR decline) and the requirement of two year’s follow-up has limited its routine implementation.

We also compared our risk score with the RENAAL progression score, which was based on a powerful and well-characterized cohort of patients with diabetic nephropathy. Similar to our study, RENAAL score included both baseline hemoglobin and serum albumin. The finding that we identify the same risk factors for progression as in the RENAAL study strongly suggests that the same factors affect nephropathy progression regardless of the original insult.

While our risk score is highly promising, it will require validation in independent cohorts. Moreover, our data is based on a retrospective chart reviews, and a prospective evaluation of this score would be useful, perhaps in more ethnically diverse patients. In addition, newer pathology classifications, as well as novel genetic and serologic markers are likely to enhance the predictive power of our risk score. For example, we have previously demonstrated that levels of galactose-deficient IgA1 are elevated in IgAN and may have a diagnostic value [35], [36]? Moreover, we have recently discovered five new genetic susceptibility loci for IgAN in a genome-wide association study (GWAS) [37]. The predictive value of both, galactose-deficient IgA1 as well as GWAS susceptibility alleles on disease progression has not yet been evaluated. Finally, the new Oxford classification of IgAN holds promise to improve risk prediction compared to the Haas grading [38]. Thus, inclusion of newer pathologic scores, and novel biomarkers may further improve the performance of the risk score and enable better risk stratification.

In summary, our new 4-variable Risk Score model is highly predictive of an individual risk of disease progression, explaining nearly 22% of the variance in outcome. In contrast with prior studies, there are three main advantages of this Risk Score: (1) the score equation is relatively simple, thus it is easy to implement in clinical practice, (2) the score has a superb sensitivity and specificity to predict ESRD when compared with other proposed scoring systems, and (3) our score is based entirely on the objective clinical variables that include routine laboratory measurements available for all newly diagnosed patients in clinical practice.

## Supporting Information

Table S1 **Unadjusted association of baseline parameters with eGFR at presentation (univariate analysis).**
(PDF)

Table S2 **Multivariate linear regression with stepwise selection for eGFR at the time of biopsy.**
(PDF)

Table S3 **Baseline characteristics of age and gender-matched healthy population controls.**
(PDF)

Table S4 **Patient characteristics by anemia and hypoalbuminemia diagnosis.**
(PDF)

Table S5 **Assessment of the predictive value of proteinuria in the risk of ESRD.**
(PDF)

Table S6 **Patient characteristics by risk score tertiles.**
(PDF)
