# Supplementary material for: Predicting Progression of IgA Nephropathy: New Clinical Progression Risk Score
Source: PLoS One. 2012 Jun 14;7(6):e38904. doi: 10.1371/journal.pone.0038904 (PMC3375310; doi:10.1371/journal.pone.0038904)
Supplement: Table S1 — Unadjusted association of baseline parameters with eGFR at presentation (univariate analysis). (PDF) [file pone.0038904.s001.pdf]

Table S1. Unadjusted association of baseline characteristics with eGFR at presentation (univariate analysis).

Variable	Univariate analysis		
	β*	95% CI	P value
Age at biopsy [year]	-1.65	-1.91, -1.39	$<2.0*10^{-16}$
Gender (reference :Male)	8.15	1.16, 15.14	0.02
Positive family history	9.33	-1.23, 19.89	0.08
Body mass index [kg/m^2]	-0.46	-1.93, 1.00	0.53
SBP [mm Hg]	-0.98	-1.14, -0.81	$<2.0*10^{-16}$
Systolic HTN	-33.41	-40.8, -26.1	$<2.0*10^{-16}$
DBP [mm Hg]	-1.24	-1.49, -0.99	$<2.0*10^{-16}$
Diastolic HTN	-27.82	-34.8, -20.8	$3.0*10^{-14}$
MAP [mm Hg]	-1.28	-1.51, -1.05	$<2.0*10^{-16}$
Pulse pressure [mm Hg]	-0.97	-1.25, -0.69	$3.0*10^{-11}$
Hypertension	-41.0	-47.3, -34.8	$<2.0*10-^{16}$
Urine protein [g/24h]	-2.34	-4.02, -0.67	0.006
Degree of proteinuria [per group]	-13.00	-17.6, -8.41	$3.8*10^{-8}$
History of gross heamaturia	13.46	4.78, 22.1	0.002
Serum UA [mg/dl]	-13.33	-15.1, -11.5	$<2.0*10^{-16}$
Hyperuricemia	-37.5	-44.0, -31.0	$<2.0*10^{-16}$
Serum albumin [g/dL]	1.94	-2.54, 6.41	0.40
Hypoalbuminemia	1.27	-7.40, 9.93	0.77
Serum triglycerides [mg/dL]	-0.04	-0.09, 0.01	0.10
Serum cholesterol [mg/dL]	-0.01	-0.09, 0.07	0.80
Hemoglobin [g/dl]	5.51	3.94, 7.08	$1.2*10^{-11}$
Anemia	29.45	22.7, 36.2	$<2.0*10^{-16}$
WBC [10^3/mm^3]	0.01	-2.12, 2.13	0.99
Serum IgA [mg/L]	-0.003	0.01, -4.07	0.08
Haas classification [per type]	-21.83	-24.8, -18.9	$<2.0*10^{-16}$

SBP: systolic blood pressure; DBP: diastolic blood pressure; MAP: mean arterial pressure; UA: uric acid; WBC: white blood cells count;
* Regression coefficient (change in eGFR per one unit change in the predictor).
