# Supplementary material for: Predicting Progression of IgA Nephropathy: New Clinical Progression Risk Score
Source: PLoS One. 2012 Jun 14;7(6):e38904. doi: 10.1371/journal.pone.0038904 (PMC3375310; doi:10.1371/journal.pone.0038904)
Supplement: Table S2 — Multivariate linear regression with stepwise selection for eGFR at the time of biopsy. (PDF) [file pone.0038904.s002.pdf]

Table S2. Multivariate linear regression with stepwise selection for eGFR at the time of biopsy (n=619).

Variable	β*	95% CI	Model R^2 (%)	P value
Age at biopsy [year]	-1.06	-1.27, -0.86	7.6	$<2*10^{-16}$
SBP [mm Hg]	-0.37	-0.51, -0.23	2.0	$2*10^{-7}$
Degree of proteinuria [per group]	-5.18	-8.47, -1.89	0.7	$2*10^{-3}$
Serum UA [mg/dl]	-8.23	-9.76, -6.70	8.3	$<2*10^{-16}$
Hemoglobin [g/dl]	2.90	1.75, 4.04	1.8	$9*10^{-7}$
Haas classification [per type]	-11.30	-13.90, -8.70	5.4	$<2*10^{-16}$
All covariates combined			54.5	$<2*10^{-16}$

SBP: systolic blood pressure; UA: uric acid

* regression coefficient (change in eGFR per one unit change in the predictor)
