# Supplementary material for: Predicting Progression of IgA Nephropathy: New Clinical Progression Risk Score
Source: PLoS One. 2012 Jun 14;7(6):e38904. doi: 10.1371/journal.pone.0038904 (PMC3375310; doi:10.1371/journal.pone.0038904)
Supplement: Table S3 — Baseline characteristics of age and gender-matched healthy population controls. (PDF) [file pone.0038904.s003.pdf]

Table S3. Baseline characteristics of age- and gender-matched healthy population controls.

	IgAN Patients N=619		Population Controls N=650		
**Variable**	**Values**	**Range**	**Values**	**Range**	**P value**
Age [years]	36.0±12.3	14-80	36.6 ± 8.3	22-50	0.33
Gender (Male :Female)	1.03:1.00		1.06:1.00		0.82
Serum creatinine [mg/dL]	1.5±1.1	0.6-7.4	0.9±0.1	0.6-1.1	$<2.2*10^{-16}$
GFR [mL/min/1.73m^2]	87.9±44.4	10.3-290.2	122.6±17.6	73.3±183.9	$<2.2*10^{-16}$
Urine Protein [g/24h]	1.42	0-13.9	Negative		
Serum Uric Acid [mg/dl]	6.5±1.7	2.6-11.3	5.5±1.3	2.7-10.0	$<2.2*10^{-16}$
Hyperuricemia (%)	256 (41.4)		71 (10.9)		$<2.2*10^{-16}$
Serum Albumin [g/dL]	3.4±0.8	0.7-5.4	4.1±0.3	3.3-5.0	$<2.2*10^{-16}$
Hypoalbuminemia (%)	128 (20.7)		0		
Serum Triglycerides [mg/dL]	175.7	42.5-1033.6	102.7	44.3-878.8	$<2.2*10^{-16}$
Serum Cholesterol [mg/dL]	198.1	32.1-469.1	179.2	103.1-312.0	$1.8*10^{-11}$
Hemoglobin [g/dl]	13.0±2.2	5.6-18.2	13.7±1.6	7.8-17.9	$<2.2*10^{-16}$
Anemia (%)	261 (42.2)		94 (14.5)		$<2.2*10^{-16}$
