# Supplementary material for: Predicting Progression of IgA Nephropathy: New Clinical Progression Risk Score
Source: PLoS One. 2012 Jun 14;7(6):e38904. doi: 10.1371/journal.pone.0038904 (PMC3375310; doi:10.1371/journal.pone.0038904)
Supplement: Table S4 — Patient characteristics by anemia and hypoalbuminemia diagnosis. (PDF) [file pone.0038904.s004.pdf]

Table S4. Patient characteristics by the diagnosis of anemia and hypoalbuminemia.

	Anemia		Hypoalbuminemia	
	**Yes N=261**	**No N=358**	**Yes N=128**	**No N=491**
**Outcomes:**				
ESRD Events (%)	51 (19.5)	16 (4.5)***	24 (18.8)	43 (8.8)**
**Baseline Characteristics:**				
Gender: Male (%)	141 (54.0)	173 (48.3)	65 (50.8)	249 (50.7)
Age at biopsy (±s.d.), [years]	37.9±12.5	34.7±12.0**	33.9±13.5	36.6±11.9*
GFR mean (±s.d.), [mL/min/1.73m^2]	70.9±45.9	100.3±38.9***	86.9±46.4	88.2±44.0
SBP mean (±s.d.), [mm Hg]	130.1±20.4	126.8±17.7*	127.1±18.0	128.5±19.2
DBP mean (±s.d.), [mm Hg]	83.1±13.7	82.1±12.4	81.9±13.9	82.7±12.7
Urine protein groups				
Mild (<1g/24h) (%)	85 (32.6)	152 (42.5)	8 (6.2)	229(46.6)
Moderate (1~3g/24h) (%)	112 (42.9)	142 (39.7)	52(40.6)	202(41.1)
Severe (>=3g/24h) (%)	64 (24.5)	64 (17.9)*	68 (53.1)	60(12.2)***
Gross hematuria (%)	53 (20.3)	72 (20.1)	19 (14.8)	106(21.6)
Serum UA mean (±s.d.), [mg/dl]	6.9±1.8	6.2±1.6***	6.6±1.8	6.5±1.7
Serum albumin mean (±s.d.), [g/dL]	3.3±0.7	3.5±0.9***	2.2±0.6	3.7±0.4***
Hemoglobin mean (±s.d.), [g/dl]	11.0±1.7	14.2±1.3***	12.3±2.5	13.0±2.0**
Haas classification				
Grade I-III (%)	37(14.2)	115(32.1)	38(29.7)	114(23.2)
Grade IV-V (%)	224 (85.8)	243(67.9)***	90(70.3)	377(76.8)
Glucocorticoid treatment (%)	143 (62.7)	150 (48.7)**	100 (84.7)	193(46.2)***

* Two-sided P<0.05
** Two-sided P<0.01
*** Two-sided P<0.001
