# Supplementary material for: Predicting Progression of IgA Nephropathy: New Clinical Progression Risk Score
Source: PLoS One. 2012 Jun 14;7(6):e38904. doi: 10.1371/journal.pone.0038904 (PMC3375310; doi:10.1371/journal.pone.0038904)
Supplement: Table S5 — Assessment of the predictive value of proteinuria in the risk of ESRD. (PDF) [file pone.0038904.s005.pdf]

Table S5. Assessment of the predictive value of proteinuria in the risk of ESRD. The best model (**model 1**) excludes proteinuria, has lowest AIC and BIC, and explains 22% of variance in the outcome. Proteinuria is not significant in the full model (**model 2**), although the effect estimate is suggestive of increased risk with higher urine protein excretion (HR 1.21, p=0.32). Exclusion of albumin in **model 3** unmasks highly significant association of proteinuria with the risk of progression (HR 1.56, p=9.7 x 10-3). Additional exclusion of hemoglobin has little effect on the strength of this association (**model 4**).

	**Selection Criteria**			**Significance of Individual Predictors in the Model (Wald test)**					
	**R2**	**AIC**	**BIC**	**eGFR P-value**	**SBP P-value**	**Hemoglobin P-value**	**Albumin P-value**	**Proteinuria # P-value**	**Proteinuria # HR (95%CI)**
**Model 1**	0.22	523.8	541.5	1.3 x 10-14 ***	5.4 x 10-3 **	1.2 x 10-5 ***	7.4 x 10-5 ***	--	--
**Model 2**	0.22	524.8	547.0	1.4 x 10-13 ***	0.012 *	1.1 x 10-5 ***	3.1 x 10-3 **	0.32 (NS)	1.21 (0.83-1.75)
**Model 3**	0.21	531.1	548.8	5.6 x 10-12 ***	0.081	7.6 x 10-7 ***	--	9.7 x 10-3 **	1.56 (1.11-2.19)
**Model 4**	0.18	552.4	565.7	2.9 x 10-13 ***	0.037 *	--	--	8.1 x 10-3 **	1.55 (1.12-2.15)

* Two-sided P<0.05
** Two-sided P<0.01
*** Two-sided P<0.001
\# Log-transformed

Model 1: predictors include baseline eGFR, systolic blood pressure (SBP), hemoglobin, albumin (the best model used for risk score derivation).
Model 2: predictors include baseline eGFR, SBP, hemoglobin, albumin, and proteinuria (proteinuria is not significant in this model).
Model 3: predictors include baseline eGFR, SBP, hemoglobin, and proteinuria (proteinuria becomes highly significant after exclusion of albumin).
Model 4: predictors include baseline eGFR, SBP, and proteinuria (proteinuria slightly more significant after exclusion of albumin and hemoglobin).
