# Supplementary material for: Predicting Progression of IgA Nephropathy: New Clinical Progression Risk Score
Source: PLoS One. 2012 Jun 14;7(6):e38904. doi: 10.1371/journal.pone.0038904 (PMC3375310; doi:10.1371/journal.pone.0038904)
Supplement: Table S6 — Patient characteristics by risk score tertiles. (PDF) [file pone.0038904.s006.pdf]

Table S6. Patient characteristics by risk score tertiles.

	Risk Sore			
	1st Tertile N=206	2nd Tertile N=206	3rd Tertile N=207	P-value
**Outcomes:**				
Risk Score Interval	< -0.89	-0.89-0.99	>0.99	
ESRD Events (%)	1 (0.5)	18 (8.7)	48 (23.2)	
HR (95%CI)	- reference -	15.3 (2.0-115.0)	79.8 (11.0-580.3)	
**Baseline Characteristics:**				
Gender: Male (%)	92 (44.7)	119 (57.8)	103 (49.8)	0.03
Age at biopsy (±s.d.), [years]	29.2±10.3	38.4±11.2	40.5±12.3	$<2*10^{-16}$
GFR mean (±s.d.), [mL/min/1.73m^2]	134.2±31.7	85.9±21.6	43.8±19.0	$<2*10^{-16}$
SBP mean (±s.d.), [mm Hg]	119.2±13.4	127.5±15.8	137.8±21.8	$<2*10^{-16}$
DBP mean (±s.d.), [mm Hg]	77.0±10.3	83.0±11.5	87.5±14.5	$<2*10^{-16}$
Urine protein groups				$2.5*10^{-13}$
Mild (<1g/24h) (%)	115 (55.8)	82 (39.8)	40 (19.3)	
Moderate (1~3g/24h) (%)	65 (31.6)	89 (43.2)	100 (48.3)	
Severe (>=3g/24h) (%)	26 (12.6)	35 (17.0)	67 (32.4)	
Gross hematuria (%)	62 (30.1)	29 (14.1)	34 (16.4)	$1.1*10^{-4}$
Serum UA mean (±s.d.), [mg/dl]	5.6±1.4	6.5±1.5	7.4±1.6	$<2*10^{-16}$
Serum albumin mean (±s.d.), [g/dL]	3.7±0.7	3.4±0.9	3.2±0.7	$2.0*10^{-13}$
Hemoglobin mean (±s.d.), [g/dl]	13.7±1.6	13.4±2.1	11.4±2.1	$<2*10^{-16}$
Haas classification				$<2*10^{-16}$
Grade I (%)	5(2.4)	9(4.4)	2(1.0)	
Grade II (%)	72(35.0)	45(21.8)	19(9.2)	
Grade III (%)	109(52.9)	89(43.2)	53(25.6)	
Grade IV (%)	17(8.3)	40(19.4)	73(35.3)	
Grade V (%)	3 (1.5)	23(11.2)	60(29.0)	
Glucocorticoid treatment (%)	67 (40.4)	90 (51.1)	136 (70.1)	$4.6*10^{-8}$
